# Phototaxis in Cyanobacteria: From Mutants to Models of Collective Behavior

**DOI:** 10.1128/mBio.02398-21

**Published:** 2021-11-23

**Authors:** Shakti N. Menon, P. Varuni, Freddy Bunbury, Devaki Bhaya, Gautam I. Menon

**Affiliations:** a The Institute of Mathematical Sciences, CIT Campus, Chennai, India; b Carnegie Institution for Science, Department of Plant Biology, Stanford, California, USA; c Homi Bhabha National Institute, BARC Training School Complex, Mumbai, India; d Departments of Physics and Biology, Ashoka University, Haryana, India; University of Washington

**Keywords:** cyanobacteria, emergent behavior, mathematical modeling, motility, phototaxis, pili

## Abstract

Cyanobacteria rely on photosynthesis, and thus have evolved complex responses to light. These include phototaxis, the ability of cells to sense light direction and move towards or away from it. Analysis of mutants has demonstrated that phototaxis requires the coordination of multiple photoreceptors and signal transduction networks. The output of these networks is relayed to type IV pili (T4P) that attach to and exert forces on surfaces or other neighboring cells to drive “twitching” or “gliding” motility. This, along with the extrusion of polysaccharides or “slime” by cells, facilitates the emergence of group behavior. We evaluate recent models that describe the emergence of collective colony-scale behavior from the responses of individual, interacting cells. We highlight the advantages of “active matter” approaches in the study of bacterial communities, discussing key differences between emergent behavior in cyanobacterial phototaxis and similar behavior in chemotaxis or quorum sensing.

## OPINION/HYPOTHESIS

Cyanobacteria comprise a diverse phylum of Gram-negative bacteria, and most are obligate photoautotrophs, although some species can grow on sugars in the dark. They can be found in marine or freshwater environments, and they can be single-celled or filamentous as well as either free-living or found in close association with other bacteria, fungi, plants, and animals (1). As photosynthetic organisms that must survive and grow in diverse and fluctuating chemical and light environments, cyanobacteria have evolved sophisticated signal transduction pathways to process and respond to various stimuli.

Over the decades, the model cyanobacterium *Synechocystis* sp. strain PCC 6803 has emerged as a powerful system to investigate phototactic behavior (2–4). Unlike the faster and more directed flagellum-driven motion of Escherichia coli, *Synechocystis* exhibits “twitching” or “gliding” motility (5). This surface-dependent motility is driven by type IV pili (T4P) that attach to the substrate and then retract to move the cell forward (6). Such motility is typically associated with the extrusion of complex polysaccharides, termed “slime,” that reduce the friction that cells experience during motion. This type of motility results in collective behavior, manifesting in the emergent shapes of cyanobacterial cell colonies exposed to light (5). This is because T4P also allows cells to attach to each other and extruded slime is a shared resource (6). The molecular and mechanical responses of individual cells to single inputs such as nutrients have been well characterized, particularly for chemotaxis (7). Notably, the state of the molecular signaling circuitry that underlies the processing of these stimuli is only predictable on average (8). Understanding the collective aspects of bacterial motility is especially important since, in the environment, bacteria are found in complex mixed communities, often in dense aggregates and stratified biofilms (9). Furthermore, organisms in their natural environment are often subject to complex, fluctuating, and spatially structured combinations of stimuli.

Several approaches have been used to model cyanobacterial phototaxis (2, 10, 11). Here, we highlight a conceptual framework, that of “active matter,” which describes the collective emergent dynamics of systems composed of self-propelled units. It has been used to describe collective behavior in systems as diverse as flocks of birds and swimming in E. coli (12). This framework suggests approaches to studying the behavior of heterogeneous bacterial colonies that are subjected to diverse light regimens and chemical stimuli. We have recently demonstrated that active matter models can recapitulate aspects of experimentally observed large-scale colony morphologies during cyanobacterial phototaxis (13, 14). Here, we propose that cyanobacteria constitute a powerful system in which to model how emergent behavior at the colony level arises from the responses of individual interacting bacterial cells to multiple stimuli.

## ADVANTAGES OF CYANOBACTERIA AS A MODEL EXPERIMENTAL SYSTEM FOR COLLECTIVE BEHAVIOR

Phototaxis can be demonstrated in a simple assay in which *Synechocystis* cells are placed as a liquid droplet on a low-concentration agarose surface. In the presence of a directional light source, usually an LED of a specific wavelength, cells typically begin to move after a few hours and can be monitored using single-particle tracking software (15). As cells move toward the droplet edge that is closest to the light source, they aggregate into groups with increasing motility and directional bias toward the light source (11). At a later stage, this leads to the formation of finger-like projections that emerge from the edge of the colony and contain hundreds of phototactic cells.

*Synechocystis* cells exhibit a range of phototactic behaviors, ranging from positive phototaxis in response to red and green light (4) to negative phototaxis under blue, UV, and high intensity light (16). The phototactic response of cyanobacterial colonies can be influenced and controlled by different wavelengths and intensities (17). Relatively few studies use combinations of wavelengths, even though these may be critical in the natural environment (see, however, reference 15). These different signals, including those mediated by small molecules such as cyclic AMP (cAMP) (18), must integrate at an individual cell level so that they feed into the motility machinery to bias motion.

The availability of several signaling and photoreceptor mutants allows for experimental investigations into how these signals are integrated (5, 19). Additionally, the ability to create targeted mutants (20) makes cyanobacteria a powerful model to test specific predictions about collective behavior. *Synechocystis* cells can also be modified to express fluorescence markers such as green fluorescent protein (GFP) or yellow fluorescent protein (YFP) (21), which could be used to monitor subpopulations of cells within a larger group. Thus, cyanobacterial phototaxis could provide an ideal system in which to model information processing and integration at the level of the single cell and the colony.

Most experimental studies of bacterial motility have focused on chemotaxis mediated by flagella. Chemotaxis involves the sensing of, and response to, small diffusible molecules, e.g., sugars or amino acids, but it is relatively challenging to precisely control the concentrations of these molecules in space and time (22). In contrast, phototaxis in cyanobacteria is a response to specific light sources whose wavelengths, intensities, and direction can be accurately controlled and combined in a time-independent manner to create complex stimulus landscapes.

Collective cellular response results from cell-cell interactions mediated by T4P and slime. Cells that lie within a neighborhood spanning a couple of cell lengths can physically attach via T4P (5, 6). This results in dense aggregates of cells whose physical connectivity is dynamic. The slime that individual cells produce facilitates the motion of cell aggregates, possibly through the reduction of friction, promotion of pilus surface attachment, or by acting as a signal to coordinate cell movement along previously laid down slime tracks (2, 6). Since slime does not diffuse or degrade quickly, it can accumulate, potentially having a significant long-term effect as a shared resource (11). While the dynamics of individual cells is governed by relatively simple rules, adding interactions between cells can lead to a variety of collective outcomes. This is most strikingly manifest in the formation of fingers, each containing hundreds of cells, that extend out of the colony toward (or away from, in the case of specific light regimes or in particular mutants) the light source. These properties of phototaxis in *Synechocystis* suggest that collective behavior in such colonies is an emergent property.

## ACTIVE-MATTER MODELS FOR COLLECTIVE BEHAVIOR IN CYANOBACTERIA

Models of collective phototaxis in cyanobacteria have incorporated many experimentally observed properties of phototactic bacteria. Such models include stochastic cellular automaton models (11), reaction-diffusion models (2), and active-matter models (13, 14, 23). Cellular automaton models assume a set of rules for determining the motion of individual cells, depending on the instantaneous properties of the underlying lattice. The responses of individual cells are incorporated as a biased random walk, where cells exhibit an increased propensity to move toward light and to traverse regions of the lattice that were previously occupied. However, these models do not account for cell-cell attachments and, hence, cannot capture macroscopic effects that are a result of aggregation. On the other hand, reaction-diffusion models attempt to capture these large-scale dynamics by modeling the change in concentrations of the cell populations as well as that of the underlying slime as a smooth field. This approach, while able to capture macroscopic morphology and dynamics of the colony, does not describe individual cell trajectories and, therefore, cannot be used to understand single-cell behavior within the colonies.

Active-matter models of phototaxis incorporate the interactions between individual cells as well as the local and long-term effects of slime. For example, the model of reference 23, where cells stochastically update their direction of motion based on the dynamics of other cells in their vicinity, shows aggregation of the type seen in cyanobacterial colonies. In the active-matter model we have recently proposed (13, 14), cells are independent agents that can sense the direction of one or more light sources. At every time step, cells move toward light with a given probability. Cells also lay down slime at their current location at a constant rate. The speed of each cell is dependent on the slime density at its current position. To model T4P attachment between cells, each cell can dynamically attach to, and detach from, a small number of its neighbors and exert forces on them. Simulations of such models can reproduce experimental observations both at the level of individual cell trajectories and the morphologies seen at the colony level. Thus, active-matter models provide a powerful framework in which several parameters can be flexibly incorporated to study emergent group behavior.

**FIG 1:**
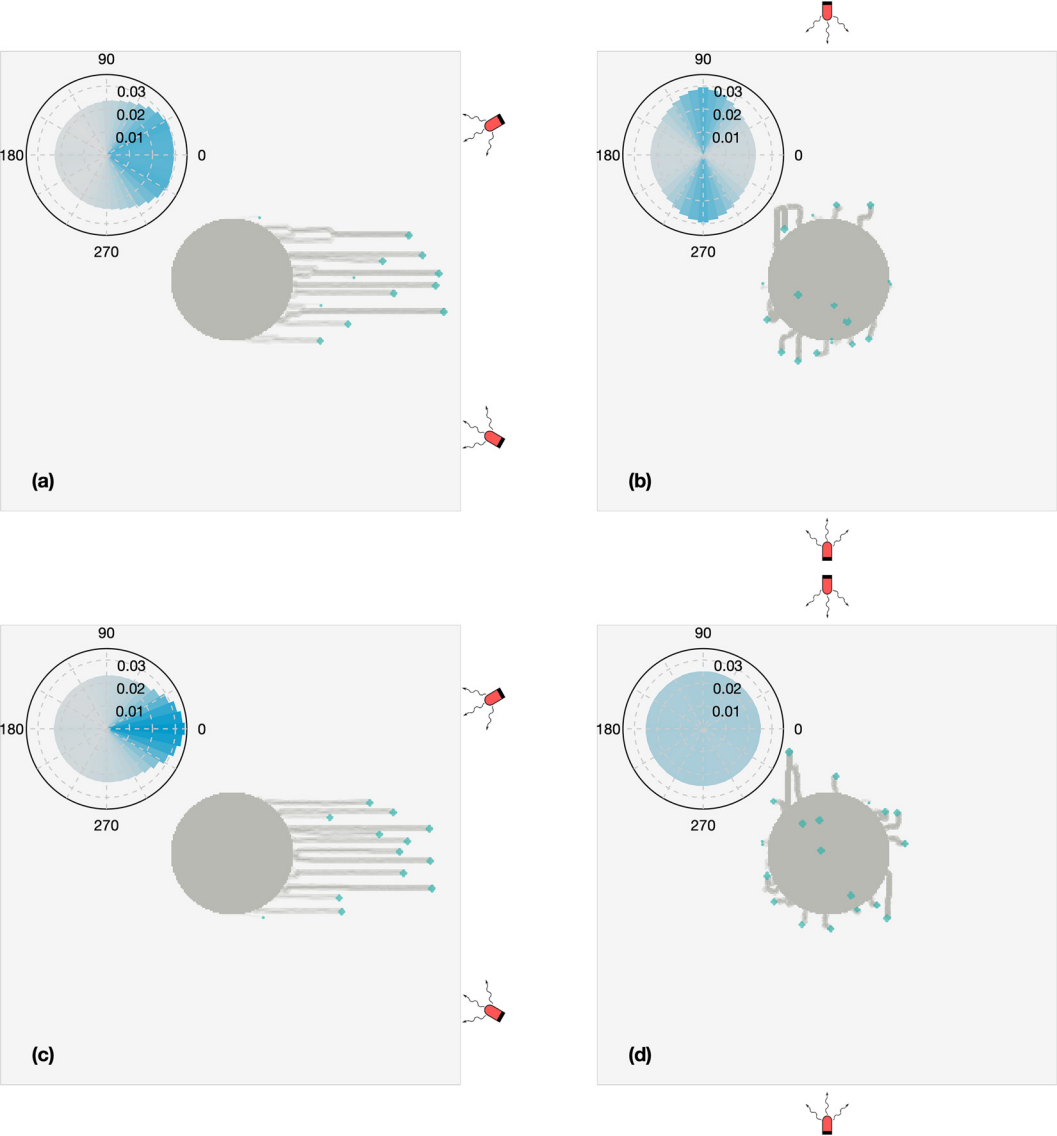
Snapshots of simulations of the model of Varuni et al. (13), adapted from reference 14. The panels display the final morphologies of colonies that are exposed to a pair of light sources placed at an angle of 30° (a and c) and 90° (b and d) north/south of east. In panels a and b, the cells detect the locations of the two light sources and stochastically bias their directions of motion toward one or the other at each time step, while in panels c and d the cells integrate the information from the two sources to determine the direction of the vector sum and bias their motion toward that computed location. The insets display the corresponding rose plots of the angle of motion in each case.

Figure 1 presents agent-based simulations of two scenarios for how decision-making at the individual cell level can affect shape changes at the colony level. In the first scenario, at every time step, each cell picks one of the light sources at random and attempts to move in that direction. In the second case, every cell computes its direction of motion as a “vector sum” based on the relative positions of the light sources with respect to the colony.

Models can suggest experimental methods of discriminating between such competing possibilities. Simulations predict that the emergent morphologies of the cell colonies should be broadly similar in both scenarios. However, the statistics of trajectories of individual cells within these colonies were significantly different in the two scenarios, suggesting that cell tracking in experiments helps differentiate between them (14).

An important feature of active-matter models is that they are agnostic to the precise mechanism by which cyanobacteria sense and respond to light. Therefore, such models allow us to propose possible hypotheses for how information integration might occur in such systems, independent of how this integration is implemented at a molecular level. However, in certain cases, colony-level behavior is insufficient to determine the underlying mechanism of information integration at an individual cell level (14, 15), and prediction of single-cell responses by subcellular mechanistic models may be necessary.

## FUTURE DIRECTIONS

Phototaxis differs from the well-studied swimming behavior of individual cells toward chemical signals, or chemotaxis. Flagellated cells exhibiting chemotaxis sense chemical gradients and move toward or away from the source of the signal via a biased random walk (8). Collective behavior in swimming chemotactic cells is a consequence of fluid-mediated hydrodynamic interactions. These interactions lead to a complex interplay between flows and swimming that is absent in phototaxis. Bacterial quorum sensing is another well-studied phenomenon in which cells coordinate their response in a density-dependent manner by producing small extracellular molecules and sensing their local concentration (24).

As with other types of T4P-mediated behavior, cyanobacterial phototaxis differs significantly from the paradigms of chemotaxis and quorum sensing in aspects of information integration and collective response. The presence of slime provides a way for cells to interact across time, since slime laid down by a cell can be used by another cell at a later time. This mechanism is analogous to the pheromones laid down by ants traversing a specific path that is sensed by ants that move along that path at a later time (25). Cyanobacteria also rely on local interactions through the physical connections of T4P and short-range effects of slime accumulation. Thus, in phototaxis, individual cells coordinate through transient physical connectivity to arrive at a consensus response in their local neighborhood that eventually leads to collective behavior. Active matter models allow the integration of single-cell behavior with the collective motion of the colony, including the effects of density and crowding.

Active-matter models are especially amenable to investigating population structure and dynamics in complex heterogeneous populations found in naturally occurring bacterial communities, such as biofilms. Tracking trajectories of cells in colonies that contain more than a single type of cell might elucidate how cells in a colony coordinate and arrive at a consensus. For example, it would be interesting to experimentally investigate, in heterogeneous populations, how a colony consisting of different light-sensing mutants might integrate information from multiple sources. Further, modeling these types of phenomena in filamentous forms of cyanobacteria that can differentiate into distinct cell types, or modeling collective behavior in other bacteria that exhibit T4P motility, such as Pseudomonas, *Neisseria*, and *Myxococcus* species, can be formulated as extensions to the framework we discuss here.

The ability to easily manipulate the signal, i.e., light, allows for detailed experimental investigations of how multiple signals are integrated within individual cells. In the natural environment, bacteria are often in dense communities or on surface-attached biofilms rather than freely swimming. It is straightforward to make the connection to models, since active-matter models allow us to vary individual cell responses and examine their consequences for resultant large-scale colony morphologies as well as provide a way to interpret experimentally observed mutant colony morphologies.

Much like the quorum-sensing system, the collective aspects of phototaxis encode a system of cell communication, although here the interactions are direct, short range, and possibly transient (17). Since heterogeneity can be easily incorporated into active matter models, exploring the dynamics of complex populations of cells in ecological contexts becomes feasible.

In conclusion, we propose that cyanobacterial phototaxis provides an ideal model system for the study of collective motion, both from an experimental and a modeling perspective. Accurately benchmarked mathematical models of such behavior can provide a way to understand these processes and interpret experimental results as well as contribute to the design and focus of future experiments.
